# Giant Scrotal Epidermoid Cyst Containing 600 g of Keratinous Material: A Rare Testis-Preserving Surgical Excision

**DOI:** 10.7759/cureus.112300

**Published:** 2026-07-08

**Authors:** Dipen K Kotwal, Nikunj Barvadiya, Anjali Aghera, Divyeshkumar N Parmar, Mohith VS

**Affiliations:** 1 General Surgery, All India Institute of Medical Sciences, Rajkot, IND

**Keywords:** epidermoid cyst, giant epidermoid cyst, giant scrotal mass, scrotum, sebaceous cyst

## Abstract

Scrotal epidermoid cysts are uncommon benign lesions and rarely attain giant dimensions. We report a 60-year-old male who presented with a progressively enlarging left scrotal swelling of one year's duration associated with a dragging sensation for two to three months. There was no associated pain, urinary complaints, fever, trauma, discharge, or constitutional symptoms. Clinical examination revealed a large, nontender cystic swelling involving the left hemiscrotum, measuring approximately 12×10 cm, with multiple sebaceous cysts over the scrotal skin. The left testis was palpable separately from the swelling. Ultrasonography, contrast-enhanced computed tomography, and magnetic resonance imaging suggested a giant epidermoid cyst arising from the left scrotal sac. Complete surgical excision was performed under spinal anesthesia. Intraoperatively, approximately 600 g of thick, whitish, pultaceous keratinous material was evacuated, followed by complete excision of the cyst wall while preserving the left testis. Redundant scrotal skin was excised, and scrotoplasty was performed. No drain was placed. The patient was discharged on postoperative day 2 with scrotal support. Histopathological examination showed an epidermoid cyst with inflammation. Giant scrotal epidermoid cysts are exceedingly rare and may mimic other benign and malignant scrotal lesions. Complete surgical excision remains the treatment of choice and provides excellent functional and cosmetic outcomes.

## Introduction

Epidermoid cysts are common benign lesions formed by the accumulation of keratin within a cyst lined by stratified squamous epithelium. They are most frequently seen on the scalp, face, neck, and trunk, whereas involvement of the scrotum is uncommon [1]. Scrotal epidermoid cysts represent a rare subset of extratesticular lesions, and the available literature consists mainly of isolated case reports and small case series [2]. In most patients, these cysts enlarge slowly and remain asymptomatic for years. Consequently, many individuals ignore the swelling until it becomes large enough to cause discomfort, interfere with daily activities, or produce an obvious cosmetic deformity. Long-standing lesions may occasionally become infected or be mistaken for other scrotal pathologies on clinical examination [3].

Giant scrotal epidermoid cysts are rarely encountered in routine surgical practice. Their presentation can resemble more common conditions such as hydrocele, spermatocele, epididymal cyst, chronic hematocele, or paratesticular tumors. We report a patient with a giant scrotal epidermoid cyst that had gradually enlarged over one year and contained approximately 600 g of keratinous material at surgery. The size of the lesion and the volume of the cyst contents make this an unusual presentation of an otherwise common pathological entity [4].

## Case presentation

A 60-year-old male presented with a gradually progressive swelling involving the left side of the scrotum for one year. The swelling was associated with a dragging sensation for the preceding two to three months. There was no history of pain, fever, trauma, discharge, ulceration, lower urinary tract symptoms, weight loss, or constitutional complaints.

The patient was a known hypertensive on regular medication. He had previously undergone an open pancreaticoduodenectomy (Whipple procedure) for carcinoma of the gallbladder and had developed a large postoperative incisional hernia. Routine hematologic and biochemical investigations were within normal limits. Two-dimensional echocardiography demonstrated a left ventricular ejection fraction of 60% with grade II diastolic dysfunction.

**Figure 1:**
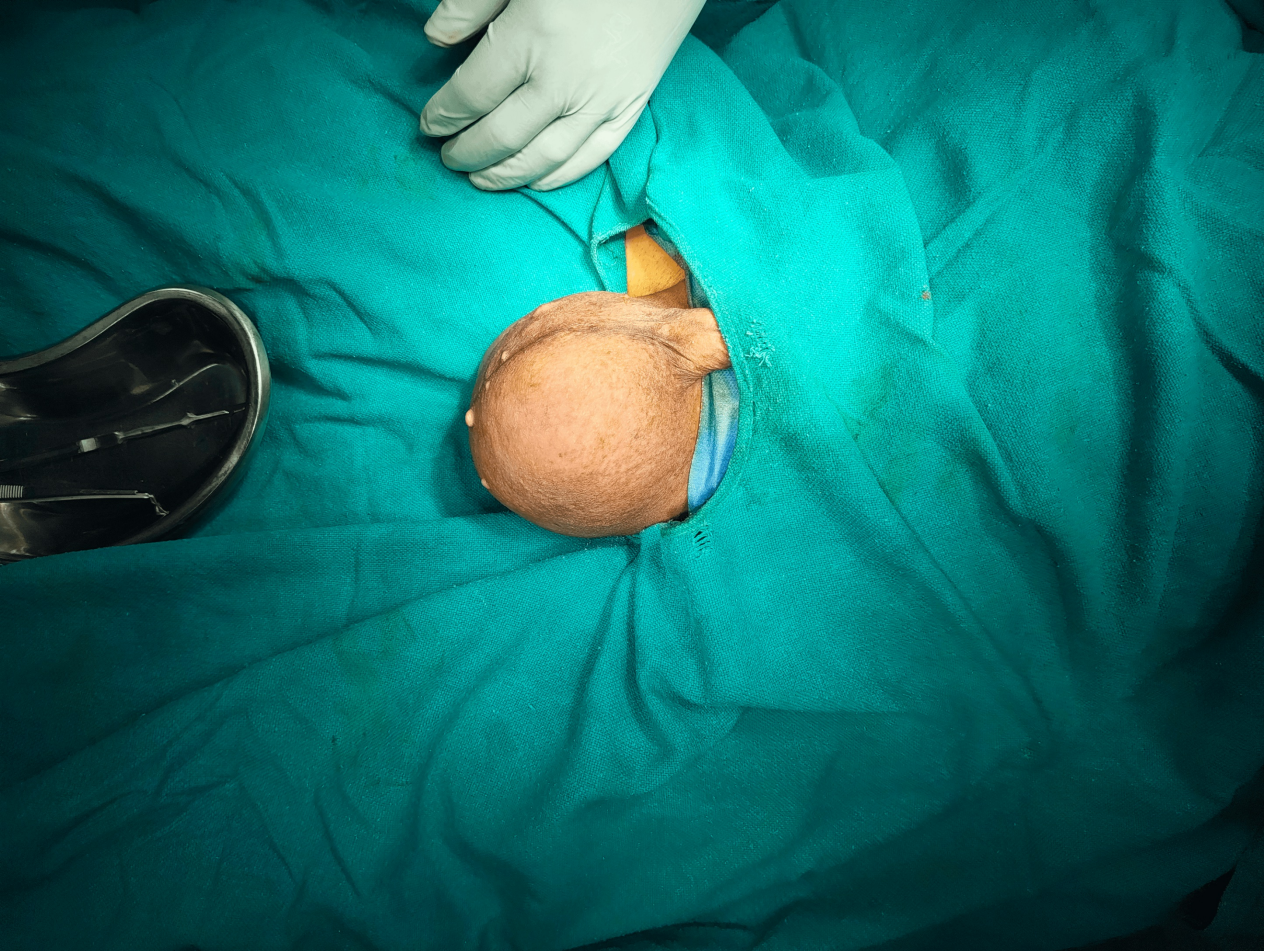
Approximately 12 × 10 cm left scrotal swelling with multiple sebaceous cysts over the scrotal wall.

On local examination, a large globular swelling involving the left hemiscrotum measuring approximately 12 × 10 cm was noted (Figure 1). The overlying scrotal skin was stretched but healthy, with multiple small sebaceous cysts scattered over the scrotal wall. No ulceration, sinus, punctum, erythema, or active discharge was present. The swelling was non-tender, cystic in consistency, smooth-surfaced, and well defined. It was nonreducible and showed no cough impulse. Transillumination was negative. The left testis was palpable separately from the swelling and appeared normal in size and consistency. The right testis and spermatic cord were unremarkable. There was no inguinal lymphadenopathy. Abdominal examination revealed a large, reducible postoperative incisional hernia along the previous upper midline laparotomy scar.

The absence of constitutional symptoms, inguinal lymphadenopathy, testicular involvement, and locally invasive features favored a benign etiology.

Ultrasonography of the inguinoscrotal region demonstrated a well-circumscribed lesion measuring approximately 11 × 10 × 10 cm arising from the left paramedian anterior scrotal wall, with mass effect on the adjacent testis, suggestive of an epidermoid cyst, with chronic encysted hydrocele considered a differential diagnosis.

**Figure 2:**
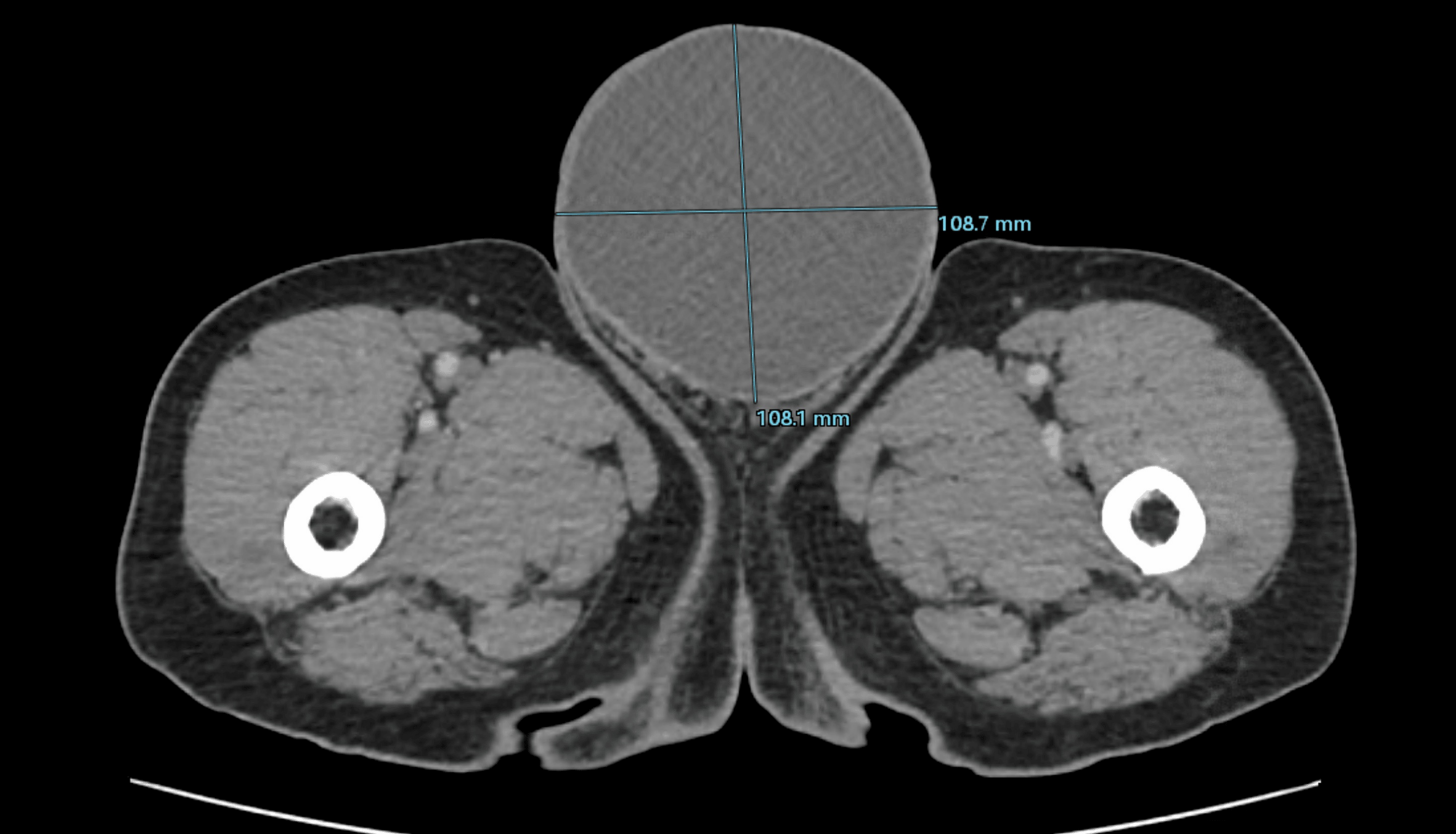
Axial computed tomography image of the scrotum demonstrating a large, well-circumscribed cystic lesion measuring approximately 10.8 × 10.9 cm, causing marked scrotal enlargement without evidence of local invasion.

Contrast-enhanced computed tomography of the abdomen and pelvis, performed during evaluation of the incisional hernia, revealed a large, rounded cystic lesion with internal septations, eccentric calcific foci, and peripheral enhancement measuring 11 × 10.8 × 10.9 cm arising from the left scrotal sac (Figure 2).

**Figure 3:**
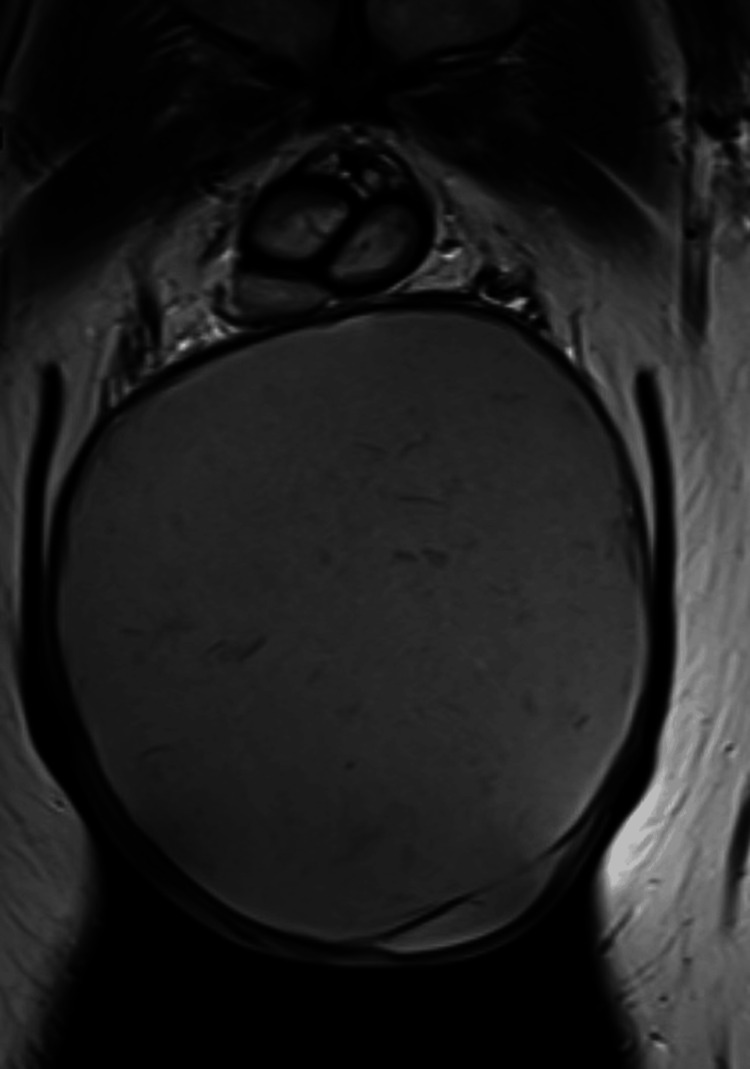
Coronal T2-weighted magnetic resonance imaging of the pelvis showing a giant, well-defined, thin-walled, homogeneous hyperintense cystic lesion occupying almost the entire scrotal sac, causing marked compression and displacement of the adjacent scrotal structures without obvious solid components or septations.

Magnetic resonance imaging demonstrated a well-defined peripherally enhancing lesion measuring 11 × 10.2 × 10.4 cm within the left scrotal sac (Figure 3).

The patient received preoperative intravenous amoxicillin-clavulanate prophylaxis and underwent surgical excision under spinal anesthesia.

**Figure 4:**
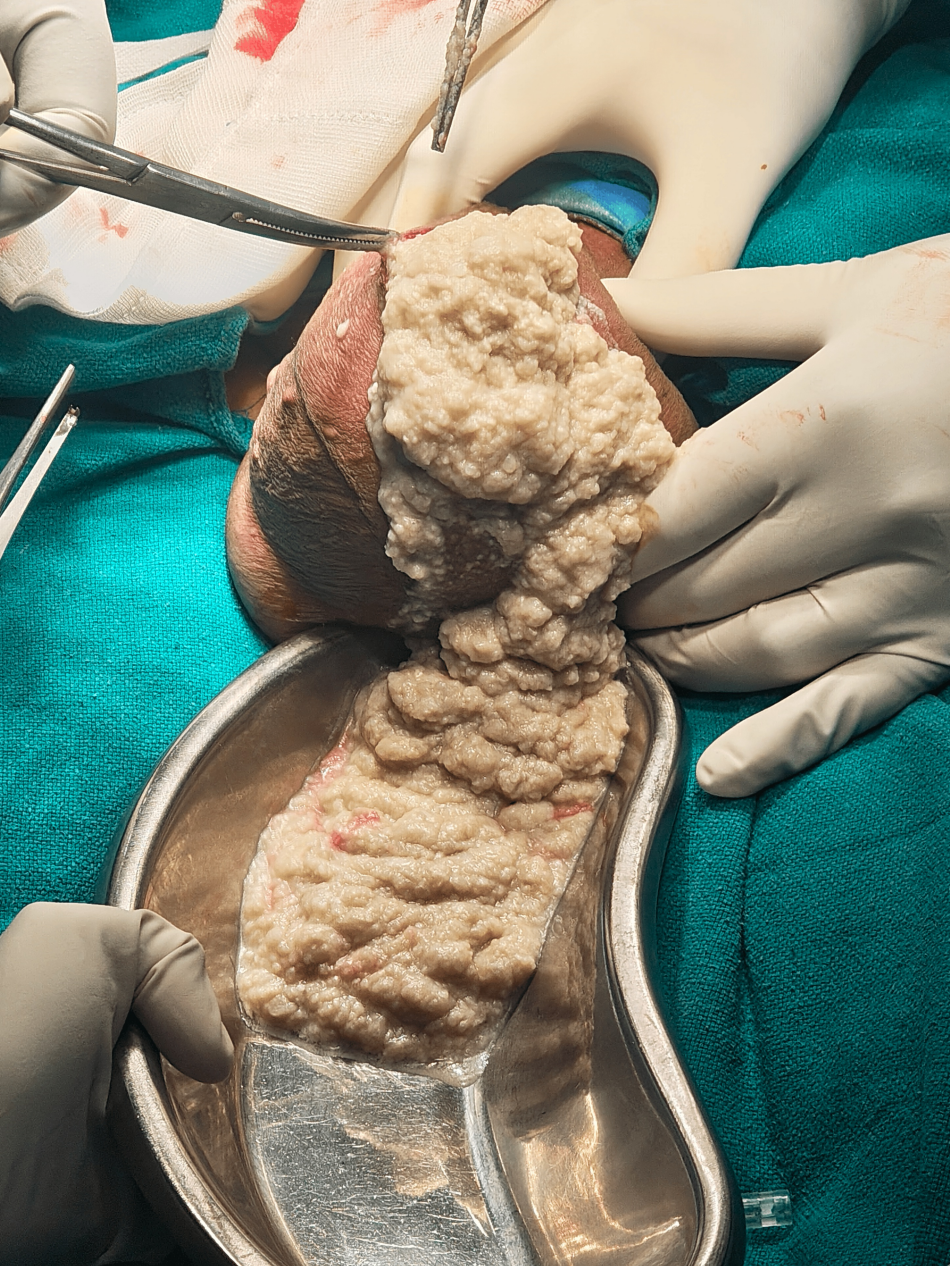
Intraoperative image showing a large amount of keratinous material evacuated from the scrotal swelling.

**Figure 5:**
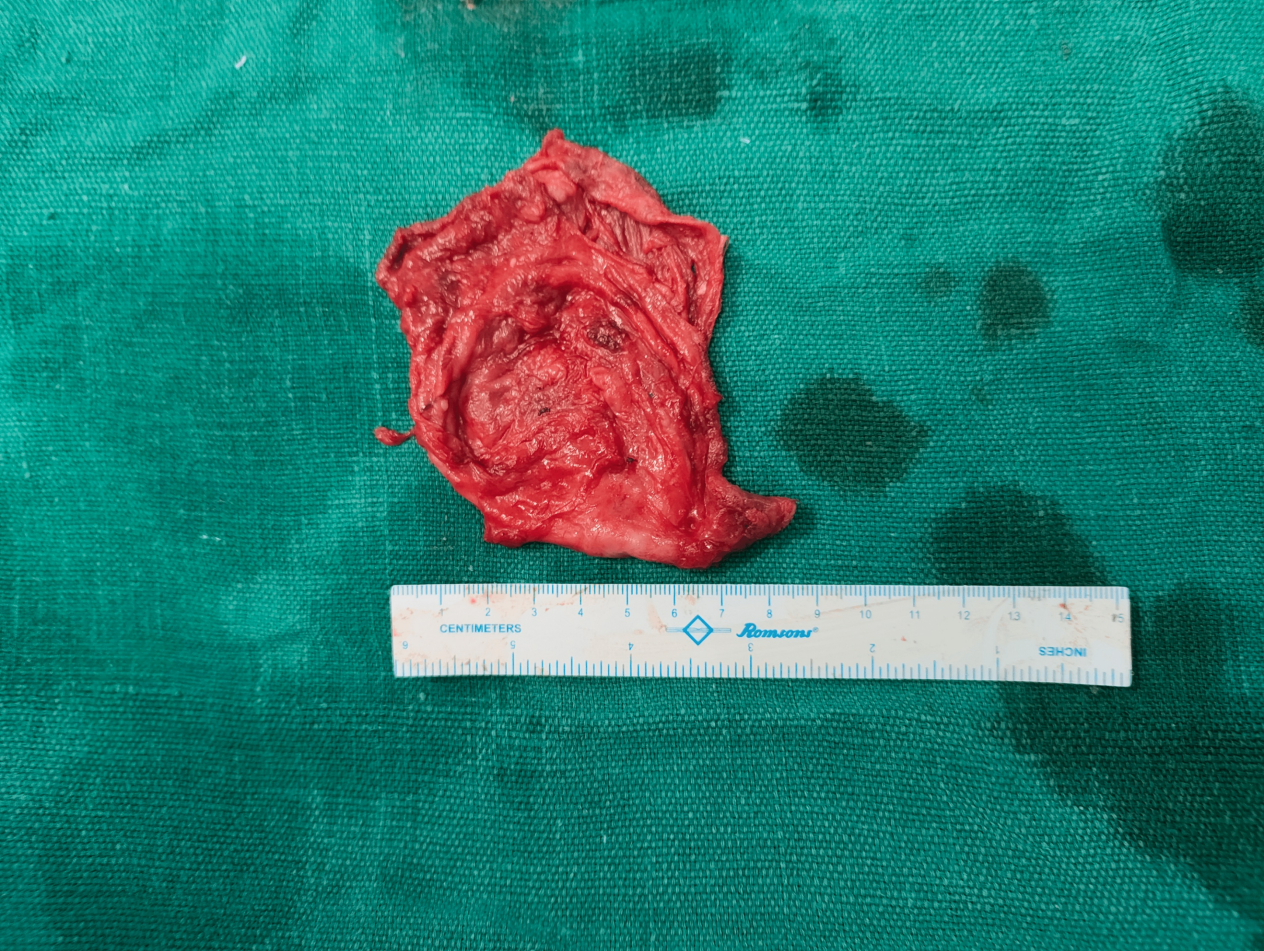
Intraoperative image showing a large amount of cyst contents evacuated from the scrotal swelling.

A vertical incision approximately 6-7 cm in length was placed 3-4 cm lateral to the median raphe. On entering the lesion, copious amounts of thick, whitish, pultaceous keratinous material were encountered and evacuated. Approximately 600 g of cyst contents were removed (Figure 4). Following decompression, the cyst wall was identified and meticulously dissected circumferentially from the surrounding scrotal tissues. The plane between the cyst wall and tunica vaginalis was carefully developed, allowing complete excision without violation of the testicular coverings. The lesion was closely related to the scrotal wall and adherent to the tunica, while the left testis was separate and viable. Complete excision of the cyst sac measuring approximately 8 × 6 cm was achieved without injury to the testis (Figure 5). The operative field was irrigated thoroughly with povidone-iodine and normal saline. Excess redundant scrotal skin was excised, and primary closure was performed. No drain was placed. Dartos closure was performed using 2-0 Vicryl, followed by skin closure with interrupted 3-0 Ethilon sutures. A sterile dressing and postoperative scrotal support were applied.

The postoperative period was uneventful, and the patient was discharged on postoperative day two. At the two-week follow-up, wound healing was satisfactory, with no evidence of infection, hematoma, seroma, wound dehiscence, or recurrence. The patient reported complete resolution of the dragging sensation and expressed satisfaction with the cosmetic outcome. Histopathology was suggestive of an epidermoid cyst with inflammation.

## Discussion

Although epidermoid cysts are among the most common benign cystic lesions encountered in clinical practice, scrotal involvement remains uncommon. Most reported scrotal epidermoid cysts are relatively small at the time of presentation. Giant lesions are unusual and are typically seen in patients who delay seeking medical attention because the swelling is painless and progresses slowly over time [5]. A similar pattern was observed in our patient, who had noticed gradual enlargement of the swelling for approximately one year before presentation.

The diagnosis may not always be straightforward, particularly when the lesion reaches a large size. Clinically, giant scrotal epidermoid cysts may resemble hydrocele, spermatocele, epididymal cyst, chronic hematocele, sebaceous cyst, scrotal abscess, or even paratesticular neoplasms. Careful examination is helpful, especially when the testis can be palpated separately from the swelling, suggesting an extratesticular origin. Ultrasonography remains the initial imaging modality of choice and typically demonstrates a well-circumscribed lesion containing internal echogenic material produced by keratin debris [6]. In selected cases, particularly when the swelling is unusually large, cross-sectional imaging can help define the extent of the lesion and its relationship to adjacent structures before surgery [7]. In our patient, computed tomography demonstrated a large cystic lesion confined to the scrotum without evidence of local invasion, findings that were later confirmed during surgery.

Despite their benign nature, giant epidermoid cysts can cause significant local problems. Progressive enlargement may lead to discomfort while walking, difficulty maintaining personal hygiene, and considerable cosmetic embarrassment. Long-standing lesions may also become infected, rupture, form chronic discharging sinuses, or undergo dystrophic calcification [8]. Although uncommon, malignant transformation arising within epidermoid cysts has been reported and should always be excluded on histopathological examination [9].

Complete surgical excision remains the treatment of choice. Simple drainage is inadequate because the epithelial lining remains behind, and recurrence may occur. In large cysts, evacuation of the keratinous contents before definitive excision can make dissection easier and reduce tension on the surrounding tissues. In the present case, approximately 600 g of keratinous material was evacuated, allowing complete removal of the cyst while preserving the ipsilateral testis. Primary closure of the redundant scrotal skin provided a satisfactory cosmetic result. The patient recovered well following surgery, and no early postoperative complications were observed.

## Conclusions

Giant scrotal epidermoid cysts are rare benign lesions that may clinically mimic a variety of cystic and neoplastic scrotal conditions. Careful clinical examination, appropriate imaging, and complete surgical excision are essential for successful management. Testis-preserving excision combined with primary closure can provide excellent postoperative recovery, satisfactory cosmetic outcomes, and a low risk of recurrence. Histopathological examination remains indispensable for the definitive diagnosis and exclusion of malignant transformation.
